# A Cross-Sectional Survey of HIV Testing and Prevalence in Twelve Brazilian Correctional Facilities

**DOI:** 10.1371/journal.pone.0139487

**Published:** 2015-10-14

**Authors:** Renata Viebrantz Enne Sgarbi, Andrea da Silva Santos Carbone, Dayse Sanchez Guimarães Paião, Everton Ferreira Lemos, Simone Simionatto, Marco Antonio Moreira Puga, Ana Rita Coimbra Motta-Castro, Mauricio Antonio Pompilio, Juliana Urrego, Albert I. Ko, Jason R. Andrews, Julio Croda

**Affiliations:** 1 University Hospital, Federal University of Grande Dourados, Dourados, Brazil; 2 Faculty of Health Sciences, Federal University of Grande Dourados, Dourados, Brazil; 3 Faculty of Ambiental and Biological Sciences, Federal University of Grande Dourados, Brazil; 4 Department of Biochemical Pharmacy, Federal University of Mato Grosso do Sul, Campo Grande, Brazil; 5 Oswaldo Cruz Foundation, Campo Grande, Brazil; 6 Faculty of Medicine, Federal University of Mato Grosso do Sul, Campo Grande, Brazil; 7 Department of Epidemiology of Microbial Disease, Yale School of Public Health, New Haven, Connecticut, United States of America; 8 Gonçalo Moniz Institute, Oswaldo Cruz Foundation, Salvador, Brazil; 9 Division of Infectious Diseases and Geographic Medicine, Stanford University School of Medicine, Stanford, California, United States of America; Johns Hopkins School of Public Health, UNITED STATES

## Abstract

**Background:**

Prior studies have reported higher HIV prevalence among prisoners than the general population in Brazil, but data have been derived from single prisons. The aim of this study was to evaluate HIV testing practices, prevalence and linkage to care among inmates in a network of 12 prisons.

**Methods:**

We administered a questionnaire to a population-based sample of inmates from 12 prisons in Central-West Brazil and collected sera for HIV and syphilis testing from January to December 2013. We evaluated factors associated with HIV testing and infection using multivariable logistic regression models. Six months after HIV testing, we assessed whether each HIV-infected prisoner was engaged in clinical care and whether they had started antiretroviral therapy.

**Results:**

We recruited 3,362 inmates, of whom 2,843 (85%) were men from 8 prisons, and 519 (15%) were women from 4 prisons. Forty-five percent of participants reported never having been tested for HIV previously. In multivariable analysis, the variables associated with previous HIV testing were lack of a stable partner (adjusted odds ratio [AOR]: 1.38; 95% CI: 1.18–1.60), completed more than four years of schooling (AOR 1.40; 95% CI: 1.20–1.64), history of previous incarceration (AOR: 1.68; 95% CI: 1.43–1.98), history of mental illness (AOR 1.52; 95% CI: 1.31–1.78) and previous surgery (AOR 1.31; 95% CI: 1.12–1.52). Fifty-four (1.6%) of all participants tested positive for HIV; this included 44 (1.54%) men and 10 (1.92%) women. Among male inmates, HIV infection was associated with homosexuality (AOR 6.20, 95% CI: 1.73–22.22), self-report of mental illness (AOR 2.18, 95% CI: 1.13–4.18), history of sexually transmitted infections (AOR 3.28, 95% CI: 1.64–6.56), and syphilis sero-positivity (AOR 2.54, 95% CI: 1.20–5.39). Among HIV-infected individuals, 34 (63%) were unaware of their HIV status; only 23 of these 34 (68%) newly diagnosed participants could be reached at six month follow-up, and 21 of 23 (91%) were engaged in HIV care.

**Conclusions:**

HIV testing rates among prison inmates are low, and the majority of HIV-infected inmates were unaware of their HIV diagnosis. Incarceration can be an opportunity for diagnosis and treatment of HIV among vulnerable populations who have poor access to health services, but further work is needed on transitional HIV care for released inmates.

## Introduction

The prison population is growing throughout the world, with an estimated populace of 10.2 million people [1]. According to the International Centre for Prison Studies (ICPS) [2], Brazil currently has the fourth largest prison population in the world (548,003 persons in penal institutions), preceded by the United States (2,228,424), China (1,701,344), and Russia (676,400). Within Brazil, the state of Mato Grosso do Sul had the highest rate of incarceration in the country, at 497 inmates per 100,000 inhabitants in 2012 [3].

Globally, prisoners have been consistently observed to have higher rates of HIV compared with the general population [4–6]. This increased prevalence has been associated with risk behaviors of inmates both prior to and during incarceration, such as intravenous drug use, needle sharing, tattooing in unsafe conditions and unsafe sexual practices [7, 8]. In the absence of public health interventions, overcrowded prisons with high rates of undiagnosed HIV infection may serve as foci for HIV transmission. On the other hand, correctional facilities could serve as sites for the timely diagnosis and treatment of HIV [9, 10]. For some inmates, arrest and introduction into a penal institution may lead to their first interaction with health services [11], representing an opportunity to provide testing to an otherwise hard-to-reach population.

In Brazil, HIV prevalence among inmates has been estimated at 3–16%, but nearly all studies have been small, single-prison investigations. We conducted a cross-sectional study in 12 Brazilian prisons to evaluate HIV testing practices among inmates, to estimate the prevalence of HIV among male and female prisoners, to assess risk factors for HIV infection, and to evaluate linkage to care among HIV-infected inmates.

## Methods

### Study setting and design

The state of Mato Grosso do Sul, located in Central-West Brazil with a population of 2.5 million people, had an inmate population of over 12,000 in 2013. The penal system is comprised of “closed” and “open” subsets to segregate high and low-risk offenders (the latter of whom are allowed to leave the prison during daytime hours); 9,913 inmates in 22 penitentiaries comprise the total closed subset. Twelve prisons were selected from a cross-sectional study performed in the population of the closed system in the 5 largest cities in the state (Campo Grande, Corumbá, Dourados, Ponta Porã and Três Lagoas) from January through December 2013 and included in the present study. The study population, 7,221 inmates, represents 59% of the total state inmate population and 73% of inmates in the closed subset. Of the 12 study prisons, eight prisons (including 6,552 inmates) housed men and four prisons (including 669 inmates) housed women.

Proportional stratified sampling was performed by using each prison as a unit of randomization. At the time of data collection, inmates were ordered numerically based on roll call lists provided by the prison administrators and a list of random numbers was generated using Epi-Info 6.04 software (Atlanta, GA, USA). The sample size was calculated using an expected 2% prevalence for HIV, with a variation of 1%, a power of 80%, and an alpha typeerror of 5%. An additional 20% individuals from each prison were included to account for anticipated loss due to refusal to participate. Only prisoners who were at least 18 years of age at time of study commencement and who consented to participate were included in the study.

### Data collection

Each participant underwent an interview utilizing a standardized questionnaire. The variables obtained during the interview included age, sex, marital status, educational attainment, drug use, sexual history, history of sexually transmitted infections (STIs), blood transfusion, tattoos, piercings, previous surgery, self-reported mental illness, previous incarceration and time in the prison. The participant’s race/ethnicity (i.e., white, black, indigenous, Asian or mixed) was self-reported.

### HIV and syphilis testing

After appropriate antisepsis, a peripheral venous blood sample (10 mL) was obtained using a vacuum tube system. Blood samples were transferred to the Laboratory of Clinical Immunology of Federal University of Mato Grosso do Sul (UFMS), processed to obtain serum, then aliquoted and stored at -20°C until the completion of serological assays.

Serum samples of participants were initially screened with a commercial enzyme linked immunosorbent assay (ELISA) for detection of antibodies against HIV-1 and HIV-2 (Murex HIV-1.2.0, DiaSorin, Italy). All positive and indeterminate specimens were confirmed by Western blot assay (Novopath HIV-I, Immunoblot, BioRad).

Syphilis exposure was tested for using a treponemal antibody ELISA (ICE* Syphilis^®^, DiaSorin, Italy), which assays for IgG or IgM to recombinant treponemal antigens; a positive test indicates prior infection with syphilis but cannot distinguish active infection from treated cases.

All prisoners with a positive HIV test underwent counseling and medical consultation after the test results and were referred for follow-up in specialized service for treatment of HIV / AIDS. Six months after diagnosis, we followed up with the prison health service, the antiretroviral therapy delivery database system (SICLOM) [12], Laboratory Test Database System (SISCEL) [13] and the specialized service for the treatment of HIV / AIDS (SAE) in each city to determine whether HIV-infected prisoners were engaged in care and whether they had started antiretroviral therapy.

### Data analysis

All questionnaires were double entered in Research Electronic Data Capture (REDCap). After entry, data were compared to search for data entry errors. SAS version 9.2 (SAS Institute, Cary, NC, USA) was used to analyze the bivariable and multivariable models. The prevalence of HIV was expressed as the percentage among inmates screened with cluster-adjusted 95% confidence-intervals. Dichotomized and categorical data were analyzed with the chi-squared test, and t-test was used to analyze continuous variables. Bivariable logistic regression was utilized to verify the association between HIV and socio-demographic factors, self-reported risk factors, and syphilis infection. Variables achieving a pre-specified level of significance (p<0.2) in bivariable analysis were included in a multivariable model, which was then trimmed using stepwise backward logistic regression and results were expressed in odds ratios (OR) and adjusted odds ratios (AOR).

### Ethical issues

All eligible participants provided written informed consent prior to study participation. The study was approved by the Research Ethics Committee at the Federal University of Grande Dourados (Number 191,877). The results of the serological tests were delivered directly to the prisoners by an infectious disease physician and the prisoners were referred for specialist treatment.

## Results

### Demographic Characteristics and Risk Behaviors

**Fig 1:**
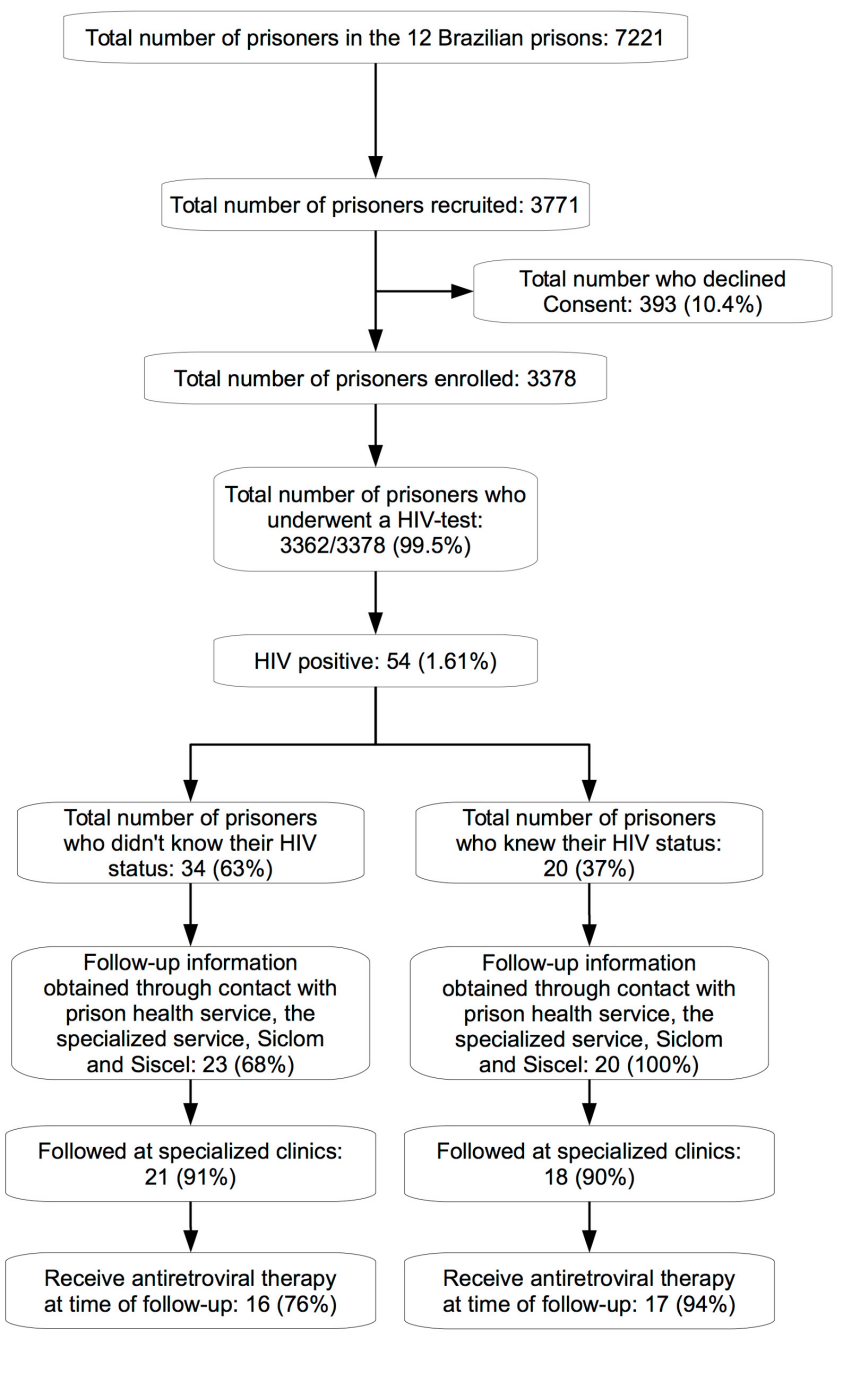
Flow chart of the study enrollment and screening process for HIV.

A total of 3,771 individuals were recruited from the 12 prisons; 393 (10.4%) individuals declined to participate in the study, and 16 (0.5%) individuals refused blood collection. Interviews were performed and blood samples collected for 3362 inmates (Fig 1). The majority of study enrolled were men (2,858; 85%), and the mean age was 32 years (SD: ±10 years, range: 18–80 years) (Table 1). Participants primarily (64%) identified as being originally from Mato Grosso do Sul, and self-reported as belonging to white (33%), mixed (51%), black (13%), indigenous (1%) and Asian (2%) ethnic backgrounds. Approximately 45% of participants reported more than four year of schooling. Drug trafficking was the most common reason for incarceration (1,525; 45%) (Tables 1 and 2).

**Table 1:** Sociodemographic and risk behaviors variables in 8 male Brazilian prisons.

Variables				Prison				
	EPC	PTL	EPRB	PHAC	CTAL	PTCG	IPCG	EPJFC
**Location**	Corumbá	TrêsLagoas	Ponta Porã	Dourados	Campo Grande	Campo Grande	Campo Grande	Campo Grande
**Prisoner population**	364	436	299	1,826	144	398	1,098	1,987
**Enrolled subjects**	263	282	252	539	116	286	517	603
**HIV positive**	4 (1.5)	5 (1.8)	3 (1.2)	10 (1.8)	3 (2.6)	5 (1.7)	8 (1.5)	6 (1.0)
**Never previously tested for HIV**	144 (54.7)	116 (41.1)	127 (50.4)	228 (42.3)	51 (44.0)	178 (62.2)	225 (43.5)	327 (54.2)
**Syphilis sero-positivity**	34 (12.9)	26 (9.2)	31 (12.3)	46 (8.5)	12 (10.3)	28 (9.8)	48 (9.3)	47 (7.8)
**Sociodemographic**								
Age, years, mean±SD	32.6 (10.0)	31.9 (10.1)	33.8 (10.7)	31.5 (9.4)	35.8 (10.1)	28.9 (9.7)	34.0 (10.0)	30.8 (8.1)
Marital status, Single	146 (55.5)	145 (51.4)	121 (48.0)	286 (53.0)	51 (44.0)	166 (58.0)	252 (48.7)	355 (58.9)
Previous incarceration	156 (59.3)	168 (59.6)	98 (38.9)	349 (64.7)	71 (61.2)	132 (46.1)	332 (64.2)	450 (74.6)
Reside in Mato Grosso do Sul	180 (68.4)	148 (52.5)	119 (47.2)	372 (69.0)	85 (73.3)	206 (72.0)	364 (70.4)	414 (68.6)
More than 4 year of schooling	111 (42.2)	124 (44.0)	144 (57.1)	199 (36.9)	59 (50.9)	134 (46.8)	190 (36.7)	241 (39.9)
Drug use over the last year	110 (41.8)	158 (56.0)	93 (36.9)	283 (52.5)	47 (40.5)	158 (55.2)	271 (52.4)	419 (69.5)
IDU over the last year	0 (0.0)	0 (0.0)	2 (0.8)	3 (0.5)	1 (0.9)	2 (0.7)	9 (1.7)	9 (1.5)
**Sexual History**								
Sexual preference, homosexual	7 (2.7)	5 (1.8)	3 (1.2)	6 (1.1)	1 (0.9)	3 (1.0)	20 (3.9)	3 (0.5)
Previously had homosexual intercourse	30 (11.4)	14 (5.0)	7 (2.8)	16 (3.0)	2 (1.7)	16 (5.6)	53 (10.2)	16 (2.6)
Ever had sex with drug user	80 (30.4)	121 (42.9)	76 (30.1)	225 (41.7)	24 (20.7)	69 (24.1)	153 (29.6)	208 (34.5)
Ever had sex with an IDU	6 (2.3)	8 (2.8)	2 (0.8)	12 (2.2)	4 (3.4)	7 (2.4)	26 (5.0)	17 (2.8)
No stable partner	143 (54.4)	140 (49.6)	125 (49.6)	274 (50.8)	41 (34.5)	130 (45.4)	245 (47.4)	317 (52.6)
Condom use, Sometimes/never	187 (71.1)	205 (72.7)	181 (71.8)	367 (68.0)	72 (62.0)	159 (55.6)	336 (65.0)	381 (63.2)
History of STI(s)	41 (15.6)	39 (13.8)	44 (17.5)	66 (12.2)	13 (11.2)	23 (8.0)	57 (11.0)	60 (9.9)
**Other Risk Behavior**								
Blood transfusion	22 (8.4)	36 (12.7)	23 (9.1)	67 (12.4)	18 (15.5)	30 (10.5)	84 (16.2)	73 (12.1)
Tattoos	171 (65.0)	191 (67.7)	111(44.0)	373 (69.2)	62 (53.4)	187 (65.4)	341 (65.9)	477 (79.1)
Piercings	27 (10.3)	25 (8.9)	7 (2.8)	22 (4.1)	109 (94.0)	253 (88.5)	485 (93.8)	541 (89.7)
Surgery	94 (35.7)	103 (36.5)	96 (38.0)	192 (35.6)	49 (42.2)	111 (38.8)	239 (46.2)	234 (38.8)
Self-reported mental illness	114 (43.3)	120 (42.5)	78 (30.9)	196 (36.4)	49 (42.2)	111(38.8)	221 (42.7)	259 (42.9)

Abbreviations: HIV- Human Immunodeficiency vírus, SD–Stardard deviation, IDU–Intravenous drug user, STI–Sexually transmitted infections, EPC—Estabelecimento Penal de Corumbá, PTL—Penitenciária de Três Lagoas, EPRB—Estabelecimento Penal Ricardo Brandão, CTAL—Centro de Triagem Anízio Lima, PTCG—Presídio de Transito de Campo Grande, IPCG—Instituto Penal de Campo Grande, PHAC—Penitenciária Harry Amorim Costa and EPJFC—Estabelecimento Penal Jair Ferreira de Carvalho.

**Table 2:** Sociodemographic and risk behaviors variables in 4 female Brazilian prisons.

Variables	Prison			
	EPFCAJG	EPFTL	EPFPP	EPFIIZ
**Location**	Corumbá	TrêsLagoas	Ponta Porã	Campo Grande
**Prisoner population**	113	82	99	375
**Enrolled subjects**	81	76	94	269
**HIV positive**	2 (2.5)	1 (1.3)	1 (1.1)	6 (2.2)
**Never previously tested for HIV**	24 (29.6)	10 (13.1)	19 (20.2)	73 (27.1)
**Syphilis sero-positivity**	8 (9.8)	14 (18.4)	11 (11.7)	55 (20.4)
**Sociodemographic**				
Age, years, mean±SD	31.8 (9.9)	31.9 (9.9)	31.9 (12.1)	31.7 (9.4)
Marital status, Single	50 (61.7)	48 (63.1)	62 (65.9)	173 (64.3)
Previous incarceration	22 (27.2)	28 (36.8)	28 (29.8)	130 (48.3)
Reside in Mato Grosso do Sul	30 (37.0)	42 (55.3)	39 (41.5)	167 (62.0)
More than 4 year of schooling	49 (60.5)	33 (43.4)	59 (62.8)	141 (52.4)
Drug use over the last year	14 (17.3)	27 (35.5)	39 (41.5)	119 (44.2)
IDU over the last year	0 (0.0)	1 (1.3)	1 (1.1)	3 (1.1)
**Sexual History**				
Sexual preference, homosexual	4 (4.9)	9 (11.8)	4 (4.2)	41 (15.2)
Previously had homosexual intercourse	19 (23.4)	19 (25.0)	20 (21.3)	67 (24.9)
Ever had sex with drug user	22 (27.2)	43 (56.6)	53 (56.4)	138 (51.3)
Ever had sex with an IDU	0 (0.0)	2 (2.6)	3 (3.2)	25 (9.3)
No stable partner	45 (55.5)	41 (53.9)	52 (55.3)	140 (52.0)
Condom use, Sometimes/never	63 (77.8)	57 (75.0)	63 (67.0)	169 (62.8)
History of STI(s)	2 (2.5)	8 (10.5)	2 (2.1)	39 (14.5)
**Other Risk Behavior**				
Blood transfusion	13 (16.0)	11 (14.5)	7 (7.4)	28 (10.4)
Tattoos	39 (48.1)	55 (72.4)	54 (57.4)	176 (65.4)
Piercings	16 (19.7)	38 (50.0)	42 (44.7)	90 (33.4)
Surgery	51 (63.0)	52 (68.4)	61 (64.9)	164 (61.0)
Self-reported mental illness	37 (45.7)	38 (50.0)	58 (61.7)	138 (51.3)

Abbreviations: HIV- Human Immunodeficiency vírus, SD–Stardard deviation, IDU–Intravenous drug user, STI–Sexually transmitted infections, EPFCAJG—Estabelecimento Penal Feminino Carlos Alberto Jonas Giordano, EPFTL—Estabelecimento Penal Feminino de Três Lagoas, EPFPP—Estabelecimento Penal Feminino de Ponta Porã, EPFIIZ—Estabelecimento Penal Feminino Irmã Irma Zorzi.

In regards to risk behaviors, half of those surveyed (1,693; 50%) reported not having a regular partner. Approximately 3% (106) of participants self-identified as homosexual, and 8% (279) reported having engaged in a homosexual intercourse. A history of any homosexual intercourse was reported more commonly among women (24%) than men (5%). Condom use was reported as irregular by 2,240 (66%) inmates, and 394 (12%) reported history of STIs. Syphilis serology was positive in 272 (9.5%) male and 88 (17%) female inmates. More than half (66%) reported having tattoos, 42% reported any mental illness and any drug use in the past year was reported among 1,738 (51%) inmates, but intravenous drug use was largely uncommon (31; 0.9%). The majority of inmates reported previous incarceration (1,964; 58%) (Tables 1 and 2).

### HIV Testing

Nearly half of inmates had never been previously tested for HIV (1,522; 45%); this ranged from 13.1% (Estabelecimento Penal Feminino de Três Lagoas) to 62.2% (Presídio de Transito de Campo Grande) among the different prisons (Tables 1 and 2). In multivariable analysis, the variables associated with previous HIV testing were lack of a stable partner (AOR: 1.38; 95% CI: 1.18–1.60), more than four years of schooling (AOR 1.40; 95% CI: 1.20–1.64), history of previous incarceration (AOR: 1.68; 95% CI: 1.43–1.98), history of mental illness (AOR 1.52; 95% CI: 1.31–1.78) and previous surgery (AOR 1.31; 95% CI: 1.12–1.52; Table 3).

**Table 3:** Variables associated with previous HIV testing among prisoners (N = 3,362).

Variables	Crude OR	Adjusted OR
Sex, female	3.18 (2.54–3.99)	
Age, per year	0.97 (0.96–0.98)	
Marital status, single	0.79 (0.69–0.90)	
Reside in Mato Grosso do Sul	0.80 (0.67–0.90)	
More than 4 year of schooling	1.40 (1.21–1.61)	1.40 (1.20–1.64)
Drug use over the last year	0.85 (0.74–0.97)	
Ever shared needles/syringes	0.91 (0.61–1.35)	
Sexual preference—homosexual	2.05 (1.34–3.14)	
Ever had homosexual intercourse	1.85 (1.43–2.41)	
Ever had sex with a drug use	1.16 (0.79–1.70)	
Ever had sex with an IDU	1.16 (0.79–1.70)	
No Stable Partner	1.35 (1,18–1,55)	1.38 (1.18–1.60)
Condom use, sometimes/never	1.07 (0.92–1.24)	
History of STI(s)	1.30 (1.03–1.59)	
Blood transfusion	1.52 (1.23–1.89)	
Tattoos	0.90 (0.78–1.04)	
Piercings	1.20 (0.97–1.49)	
Shared objects	1.10 (0.74–1.63)	
Surgery	1.59 (1.38–1.83)	1.31 (1.12–1.52)
Self-reported mental illness	1.58 (1.37–1.82)	1.52 (1.31–1.78)
Previous incarceration	1.40 (1.23–1.62)	1.68 (1.43–1.98)
Time in prison, per month	1.00 (0.99–1.00)	

Abbreviations: OR—odds ratio, IDU—intravenous drug user, STI—sexually transmitted infections.

### HIV Prevalence, Risk Factors and Linkage to Care

Among the 3362 participants, 54 (1.61%, 95% CI: 1.21%-2.10%) tested HIV-positive: 44 (1.54%; 95% CI: 1.12%-2.07%) men and 10 women (1.92%; 95% CI: 0.92%-3.53%). In bivariable analysis, male inmates who were single (OR 1.93, 95% CI: 1.02–3.64), identified as homosexual (OR: 8.39, 95% CI: 3.15–22.34), reported no stable partner (OR: 2.05, 95% CI: 1.10–3.84), had a history of STIs (OR: 3.68, 95% CI: 1.95–6.94), had positive syphilis serology (OR 3.61, 95% CI: 1.84–7.08) or had self-reported mental illness (OR: 2.26, 95% CI: 1.23–4.17) were more likely to be infected with HIV (Table 4). Among female inmates, only self-reported history of STIs was associated with HIV (OR: 30.27, 95% CI: 5.93–154.38). In multivariable analysis, homosexuality (AOR: 6.20, 95% CI: 1.73–22.22), history of STIs (AOR: 3.28, 95% CI: 1.64–6.56), positive syphilis serology (AOR: 2.54, 95% CI: 1.20–5.39) and history of mental illness (AOR: 2.18, 95% CI: 1.13–4.18) were positively associated with HIV infection among men. There were too few female HIV-infected inmates for multivariable analysis to provide reliable risk factor estimates.

**Table 4:** Risk factors associated with HIV among male and female prisoners (N = 3,362).

	Male (N = 2,843)		Female (N = 519)
Variables	Crude OR	Adjusted OR	Crude OR
Age, per year	0.98 (0.95–1.00)		0.97 (0.92–1.02)
Marital status, single	1.93 (1.02–3.64)		4.79 (0.60–38.14)
Reside in Mato Grosso do Sul	1.30 (0.71–2.38)		0.76 (0.21–2.71)
More than 4 year of schooling	0.64 (034–1.22)		0.35 (0.09–1.37)
Drug use over the last year	1.03 (0.57–1.85)		0.93 (0.26–3.35)
Ever shared needles/syringes	0.61 (0.14–2.57)		0
Sexual preference—homosexual	8.39 (3.15–22.34)	6.20 (1.73–22.22)	1.97 (0.41–9.52)
Ever had homosexual intercourse	2.42 (0.94–6.26)		0.93 (0.18–4.65)
Ever had sex with a drug use	0.75 (0.42–1.38)		0.99 (0.28–3.45)
Ever had sex with an IDU	0.84 (0.11–6.18)		4.06 (0.82–20.04)
No Stable Partner	2.05 (1.10–3.84)		2.06 (0.53–8.04)
Condom use, sometimes/never	0.53 (0.29–0.95)		1.63 (0.33–7.95)
History of STI(s)	3.68 (1.95–6.94)	3.28 (1.64–6.56)	30.27 (5.93–154.38)
Syphilis-positive	3.61 (1.84–7.08)	2.54 (1.20–5.39)	3.36 (0.93–12.18)
Blood transfusion	1.15 (0.45–2.94)		0.29 (0.07–1.16)
Tattoos	0.46 (0.25–0.83)		0.59 (0.17–2.07)
Piercings	0.99 (0.30–3.21)		5.19 (0.65–41.26)
Shared objects	0.66 (0.34–1.29)		0.93 (0.27–3.27)
Surgery	1.06 (0.58–1.95)		0
Self-reported mental illness	2.26 (1.23–4.17)	2.18 (1.13–4.18)	0.91 (0.26–3.18)
Previous incarceration	1.09 (0.59–1.98)		0.66 (0.19–2.32)
Time in prison, per month	1.01 (0.99–1.03)		0.98 (0.95–1.01)

Abbreviations: OR—odds ratio, IDU—intravenous drug user, STI—sexually transmitted infections.

Among the 54 individuals who tested positive for HIV, 34 (63%) were unaware of their diagnosis, and among these, 15 (44%) had never been previously tested for HIV. All 34 individuals who were diagnosed with HIV underwent medical examination and were referred to the HIV/AIDS referral service. After 6 months, 23 could be contacted for follow up: 21 of 23 (91%) individuals contacted had been seen in an HIV specialty clinic and 16 (76%) had started antiretroviral therapy. Among the 20 individuals who already knew their diagnosis, 18 (90%) were being followed in HIV specialized clinics and 17 (85%) were receiving antiretroviral therapy (Fig 1).

## Discussion

Globally, prison inmates are a consistently high-risk and yet underserved population for HIV, despite years of emphasis on the need for increased HIV testing and prevention in this setting [14]. Critically, nearly half of individuals in this high-risk population had never been tested for HIV, and the majority of individuals identified with HIV were not aware of their HIV status. These findings underscore the continued gap in HIV services for this population.

Prior studies in Brazil have reported HIV prevalence ranging from 1.2–16.0% in men [9, 15–20] and 9.9–13.9% in women [7, 21]. In this study of twelve prisons in Mato Grosso do Sul, we detected HIV infection among 1.54% of male inmates and 1.92% of female inmates, which while lower than reports from larger urban areas, is nevertheless 4–5 times the HIV prevalence in the general population (0.4%) [22]. The low HIV prevalence in prisons compared to others studies may be related to the decrease in intravenous drug use [23]. In 2006–2007, a survey was conducted in three state prisons that were also included in our study (Estabelecimento Penal Feminino Irmã Irma Zorzi, Instituto Penal de Campo Grande and Estabelecimento Penal Jair Ferreira de Carvalho); this study found that 10% of inmates were IDUs, and overall prevalence was 5% [24]. Similar prevalence of IDU and HIV was found in other Brazilian prisons during the last decade [7, 17]. These data may be in contrast to other prison settings, where injection drug use remains an important factor in HIV infection among inmates [25]. Declining HIV infection rates have been reported in some penal systems [26, 27]; however, global data are sparse, and better, representative data are needed to understand trends in disease burden in prisons and their drivers.

In the 2013 UNODC *World Drug Report*, 14.0 million people between the ages of 15 and 64 were estimated to be injecting drugs, while 1.6 million people who inject drugs were estimated to be living with HIV. This reflects a 12% decline in the number of people who inject drugs and a 46% decline in the number of people who inject drugs that are living with HIV since the 2008 estimates [28, 29].

An increasing number of countries have introduced HIV programmes in prisons since the early 1990s, but many of them are small in scale, restricted to a few prisons. Despite WHO and the Ministry of Health of Brazil recommend HIV testing for all prisoner at the time of incarceration for over 10 years [4, 30, 31], several studies among prisoners reported low testing rates (35%-84%) [17, 32, 33] and lack of awareness of HIV status among infected individuals (20%-65%) [8, 32, 34]. These findings demonstrate low adherence to these recommendations and critical need to implement routine testing programs in prison environments. The difficulty related to implementing such programs maybe in part due to the stigma associated with HIV status in the prisons. Human rights and privacy need to be protected, and policies against discrimination on the basis of HIV status need be monitored and enforced; a recent study highlighted the need for such provisions for prisoners in Brazil [35].

In addition to early diagnosis and treatment, other preventive measures are necessary to achieve more effective control of HIV in prisons. These include: prevention and treatment of STIs, treatment of drug dependence, needle and syringe exchange programs, condom distribution, health education, prevention of sexual violence, provision of safe practices for tattooing and piercing, and availability of post-exposure prophylaxis for HIV [4].

The results of this study have several limitations. Ten percent of inmates declined to participate in the study, and it’s possible that this group was at differential risk for HIV. We had limited access to clinical data on HIV-infected individuals, particularly with regard to the level of CD4, other co-infections and antiretroviral resistance. We performed serologic testing for syphilis but were unable to perform RPR or FTA-ABS to confirm active cases. While all HIV-infected participants were informed of their diagnosis, we were unable to reach 34% of those with a new HIV diagnosis at the time of attempted follow up six months after the initial survey. The short duration of incarceration was the primary reason for this challenge, which both limits the interpretation of linkage to care results and highlights the important area of transitional medical care that merits further work. Finally, the findings of present study may not be generalizable to other epidemiological situations, as the prevalence of injecting drug use was very low in our study in comparison with elsewhere in Brazil.

## Conclusion

In Brazil, it is estimated that approximately 718,000 people are living with HIV/AIDS, among whom 20% are currently undiagnosed [36]. Despite the recognition of prison inmates as a high-risk population, we found an even higher proportion of undiagnosed HIV in this group. These results, along with prior studies from Brazil, indicate that HIV testing should be offered upon admission to all prisoners. Incarceration provides an opportunity for diagnosis and treatment of HIV and other STIs among this vulnerable population who often has poor access to health services outside of prison. Given the high rates and short duration of incarceration, and the high proportion of undiagnosed HIV, interventions among inmates may in fact reach a considerable portion of the underserved HIV-infected population in Brazil.

## Supporting Information

S1 File Complete dataset. (CSV)
